# Differences in Complexion between Cold- and Heat-Prescription Groups in Sasang Medicine

**DOI:** 10.1155/2017/9701978

**Published:** 2017-08-14

**Authors:** Young Joo Park, Jun-Hyeong Do, Honggie Kim, Jong Yeol Kim

**Affiliations:** ^1^KM Fundamental Research Division, Korea Institute of Oriental Medicine (KIOM), 1672 Yuseong-daero, Yuseong-gu, Daejeon, Republic of Korea; ^2^Korean Medicine Life Science, University of Science and Technology, 217 Gajeong-ro, Yuseong-gu, Daejeon, Republic of Korea; ^3^Department of Information and Statistics, Chungnam National University, 99 Daehak-ro, Yuseong-gu, Daejeon, Republic of Korea

## Abstract

Sasang constitutional medicine (SCM) is a type of traditional Korean medicine (TKM) that classifies the human constitution into four types. The appearance of the complexion is one of the diagnostic factors of SCM but is rarely used in a quantitative and objective manner for diagnosis. In this study, an analysis using actual clinical data was conducted to assess the use of the complexion as a diagnostic element. A total of 528 Sasang medicine prescriptions from Korea Constitutional Multicenter Bank (KCMB) were classified into either a Cold-prescription group or a Heat-prescription group, and the complexion differences of the patients were analyzed using the *L*^⁎^*a*^⁎^*b*^⁎^ color space. After adjusting for age, BMI, and systolic blood pressure, significant differences were observed among the Cold- and Heat-prescription groups within each Sasang constitution. However, when the Sasang constitution was ignored, no significant difference was observed for either sex. This study quantitatively analyzed the complexion of patients, which is used as a diagnostic element in clinical practice. It is hoped that the results will contribute to objective medical treatments in the future, such as determining an appropriate herbal prescription based on the patient's complexion.

## 1. Introduction

In traditional Korean medicine (TKM), facial observation is one of the four examinations, and it is a factor in disease diagnosis [1]. In “The Treasure of Oriental Medicine” [2], numerous phrases are used to diagnose diseases and prescribe treatment according to patient's complexions, such as yellowish complexion, blue complexion, dark and blue complexion, white and dirty complexion, silver complexion, and red and shiny complexion. Furthermore, it is known that the complexions of each facial area reflect the state of the five viscera. It is also believed that the diseases of these five viscera and the complexions are related to each other.

The groups of disease in TKM can be divided into Cold and Heat patterns. Facial color, in addition to Cold and Heat, thirst, bowel and bladder function, tongue coat, and pulse, is an important differentiating factor in distinguishing these two patterns [1]. Moreover, in “The Yellow Emperor's Canon Internal Medicine” [3], it is stated that the complexion and Cold-Heat pattern are closely related (“when a saint looks at a person's complexion, the yellow and red color sees it as having a lot of heat, while the blue and white color means that it has less heat, and the black color has a lot of blood and a little energy”).

In Lee's book, “Longevity and Life Preservation in Oriental Medicine” [4], it is stated that the severity and the pattern changing of diseases are determined through the complexion. In the case of the So-Eum type, a blue complexion primarily appears when the conditions of the illness are severe or worsened, and yellow and red are expressed as a complexion when the illness is not severe or is subsiding. Lee claimed that the complexion reflects both the Sasang constitution and the state of the body.

In modern times, various elements related to the complexion have been studied. One of the elements is blood pressure. Skin color has been associated with both the systolic and diastolic blood pressure [5]. Specifically, a dark skin color is known to be associated with high blood pressure [6]. Additionally, with aging, the complexion tends to become dark, red, and yellow [7]. In medical studies, a difference in the complexion has been observed according to the degree of health; compared to the healthy group, the disease group was darker, and the subhealthy group was more pale [8]. Another study also examined the relationship between diseases and the complexion and showed that, in kidney disease, the complexion appeared dark, whereas in the case of hypertension or heart failure, a dark red color appeared [9].

While the relevance of the complexion to diseases in Western medicine is a subject that has been studied recently, the complexion is an essential diagnostic factor in TKM for diagnosing a disease and determining the prescription. Attempts have been made to quantitatively analyze the complexion to use this important factor objectively in TKM diagnoses [10]. One study examined the use of the complexion as a diagnostic element in SCM, in addition to body shape or symptoms [11, 12]. Although some studies have examined the use of the complexion as a diagnostic factor, objective evidence based on clinical data has been insufficient. In this study, we used objective evidence based on actual clinical data so that the complexion could be used in the objective diagnosis and treatment of SCM.

## 2. Materials and Methods

### 2.1. Data and Classification

Sasang medicine prescriptions collected by the Korea Constitutional Multicenter Bank (KCMB) were analyzed in this study. The data cases were taken, primarily the symptoms and progress of those patients that were given Sasang herbal medicine by doctors in Korean medicine clinics from 2013 to 2015. Diagnosis of Sasang type was performed by an expert using Sasang Constitution Analysis Tool (SCAT) [13]. Among the 915 subjects, data on 551 Tae-Eum-type, So-Eum-type, and So-Yang-type patients who were prescribed Taeumjowi-tang, Choweseuncheng-tang, Galgeunhaegi-tang, Yuldahanso-tang, Bojungikgi-tang, Jeokbaekhaogwanjung-tang, Gwankeibujalijung-tang, Baekhaolijung-tang, Hyangsayangwi-tang, Palmulgunja-tang, Hyungbangsabaek-san, Hyeongbangdojeok-san, Hyeongbangjihwang-tang, and Yanggyeoksanhwa-tang were selected. As 23 cases of complexion data failed to be extracted from the image, analysis was performed using data from 528 cases. Tae-Yang-type patients were excluded due to lack of subjects. Each Sasang prescription was classified into either a Cold- or Heat-prescription group (Table 1). Classification of Cold- and Heat-prescription group was based on “Longevity and Life Preservation in Oriental Medicine” [4].

### 2.2. General Characteristics of Subjects

Some significant differences were observed in the general characteristics of female (Table 2). For the So-Eum type, age of the Cold-prescription group was higher than that of the Heat-prescription group (*p* = 0.008), whereas weight of the Heat-prescription group was higher than that of the Cold-prescription group (*p* = 0.005). For the So-Yang type, age of Heat-prescription group was higher than that of the Cold-prescription group (*p* = 0.002). But no significant differences were observed in other characteristics or in males. BMI and age were adjusted in the analysis.

### 2.3. Photo Processing

#### 2.3.1. Facial Photography

**Figure 1:**
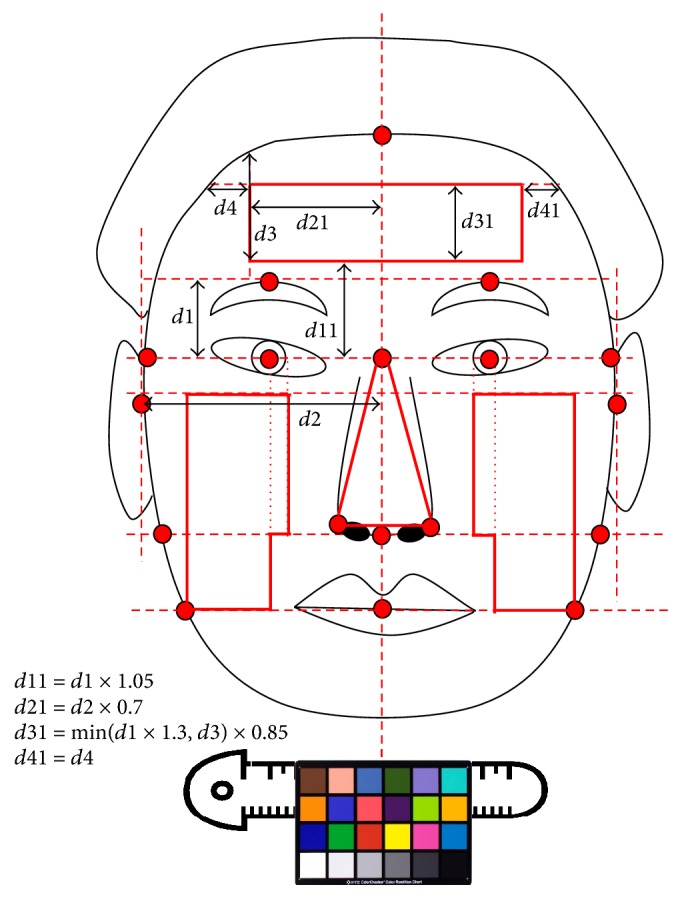
Facial complexion regions for the forehead, cheek, and nose. The regions are defined by the facial landmarks.

A frontal face photograph was obtained according to a Standard Operation Process (SOP). Then, a color chart was attached to a reference ruler for color correction and placed approximately 1 cm below the subject's chin (Figure 1) [14].

#### 2.3.2. Color Correction Process

1. The position of the color chart was automatically recognized in the image, and the color information of each cell in the color chart was extracted.
2. A color conversion model was generated by analyzing the relationship between the extracted color information and the reference color information of the color chart.
3. The generated conversion model was applied to the input image to convert the color of the input image.

#### 2.3.3. Detection of the Complexion Regions

1. Facial landmarks used to define the complexion regions were detected automatically using an image processing technique (Figure 1).
2. Three complexion regions (forehead, cheek, and nose) were defined using the detected facial landmarks. And total is the average *L*^*∗*^*a*^*∗*^*b*^*∗*^ value calculated using the *L*^*∗*^*a*^*∗*^*b*^*∗*^ value of three regions (forehead, cheek, and nose).

#### 2.3.4. Extraction of Three Variables in the Complexion Regions

**Figure 2:**
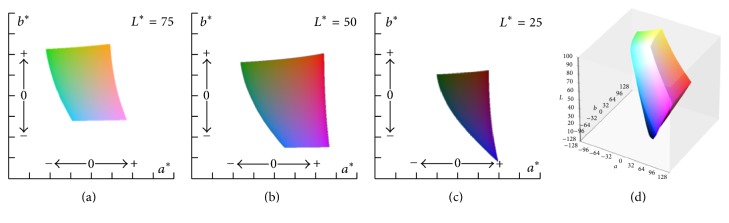
*L* ^*∗*^ *a* ^*∗*^ *b* ^*∗*^ color space [15]. (a) sRGB gamut when *L*^*∗*^ value is 75. (b) sRGB gamut when *L*^*∗*^ value is 50. (c) sRGB gamut when *L*^*∗*^ value is 25. (d) sRGB gamut within *L*^*∗*^*a*^*∗*^*b*^*∗*^ color space.

1. The red (R), green (G), and blue (B) values of each pixel in the complexion region were extracted.
2. Because the R, G, and B values are sensitive to illumination changes, these values were converted into *L*^*∗*^ values for lightness and *a*^*∗*^ and *b*^*∗*^ values for color-opponent dimensions (Figure 2) [15].
3. The mean value of *L*^*∗*^, *a*^*∗*^, and *b*^*∗*^ in each pixel in the complexion region was calculated.

### 2.4. Statistical Analysis

The relationships of the complexion with age, BMI, and systolic blood pressure were analyzed using Pearson's correlation coefficient. The *L*^*∗*^*a*^*∗*^*b*^*∗*^ components of each facial region were analyzed using analysis of covariance (ANCOVA), and the age, BMI, and systolic blood pressure were used as covariates. SPSS 20 software (IBM Corp., Armonk, NY, USA) was used for the statistical analyses.

### 2.5. Institutional Review Board (IRB)

This study was approved by the IRB of the Korea Institute of Oriental Medicine (I-1210/002-002-03).

## 3. Results

### 3.1. The Relationships of the Complexion with the Age, BMI, and Systolic Blood Pressure

**Figure 3:**
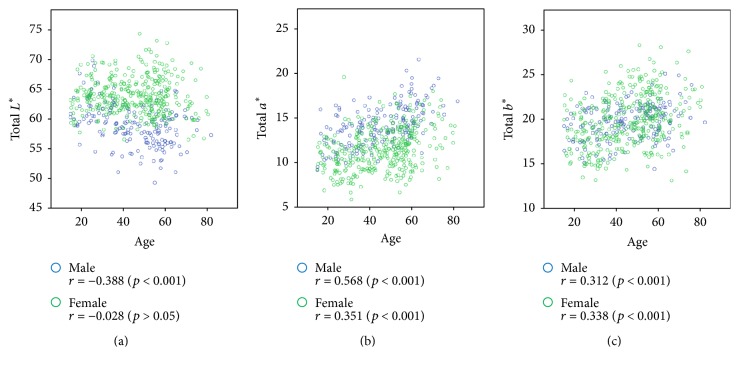
Scatter plot of the *L*^*∗*^*a*^*∗*^*b*^*∗*^ values versus age. Pearson correlation coefficients for associations of total *L*^*∗*^, total *a*^*∗*^, and total *b*^*∗*^ values with age are presented in the figures with *p* value. (a) Scatter plot of the total *L*^*∗*^ value versus age. (b) Scatter plot of the total *a*^*∗*^ value versus age. (c) Scatter plot of the total *b*^*∗*^ value versus age.

Figure 3 shows the relationship between the value of *L*^*∗*^*a*^*∗*^*b*^*∗*^ in complexion and the age. In males, the age and *L*^*∗*^ values showed a negative correlation, indicating that the complexion darkens as the age increases. The age and both *a*^*∗*^ and *b*^*∗*^ values were positively correlated in both sexes, which indicates that, with increasing age, the complexion becomes more red and yellow.

**Figure 4:**
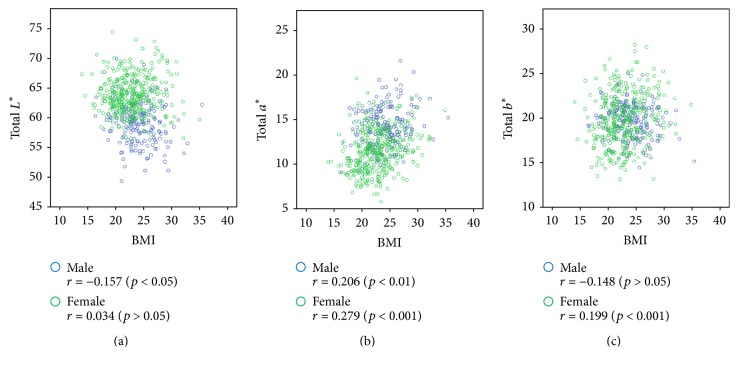
Scatter plot of the *L*^*∗*^*a*^*∗*^*b*^*∗*^ values versus BMI. Pearson correlation coefficients for associations of total *L*^*∗*^, total *a*^*∗*^, and total *b*^*∗*^ values with BMI are presented in the figures with *p* value. (a) Scatter plot of the total *L*^*∗*^ value versus BMI. (b) Scatter plot of the total *a*^*∗*^ value versus BMI. (c) Scatter plot of the total *b*^*∗*^ value versus BMI.

Figure 4 shows the relationship between the value of *L*^*∗*^*a*^*∗*^*b*^*∗*^ in complexion and the BMI. In males, the BMI and *L*^*∗*^ values showed a negative correlation, indicating that as the BMI increases, the complexion becomes darker. In both men and women, a positive correlation was observed between the BMI and *a*^*∗*^ value, indicating that as the BMI increases, the complexion tends to become more complex. In addition, a positive correlation was found between the BMI and *b*^*∗*^ values in females, indicating that as the BMI increases, the complexion becomes increasingly associated with yellow.

**Figure 5:**
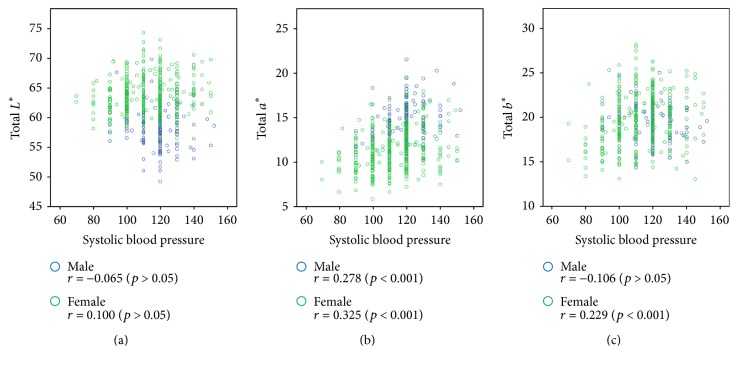
Scatter plot of the *L*^*∗*^*a*^*∗*^*b*^*∗*^ values versus systolic blood pressure (SBP). Pearson correlation coefficients for associations of total *L*^*∗*^, total *a*^*∗*^, and total *b*^*∗*^ values with SBP are presented in the figures with *p* value. (a) Scatter plot of the total *L*^*∗*^ value versus SBP. (b) Scatter plot of the total *a*^*∗*^ value versus SBP. (c) Scatter plot of the total *b*^*∗*^ value versus SBP.

Finally, we examined the relationship between the systolic blood pressure and the value of *L*^*∗*^*a*^*∗*^*b*^*∗*^ in complexion in Figure 5. The systolic blood pressure and *a*^*∗*^ values were positively correlated in both males and females; thus, as the systolic blood pressure increased, the complexion became redder.

### 3.2. Analysis of Facial Complexion according to Cold- and Heat-Prescription Groups in Each Sasang Type

Table 3 shows the facial complexion analysis for males. For the Tae-Eum type, the *L*^*∗*^ value of the cheek and the *a*^*∗*^ value of the whole face, forehead, and nose of the Heat-prescription group were larger than those of the Cold-prescription group (*p* = 0.018, 0.045, 0.016, and 0.019, resp.). For the So-Eum type, the *b*^*∗*^ value of the whole face and forehead of the Heat-prescription group was larger than that of the Cold-prescription group (*p* = 0.049, 0.018, resp.). For the So-Yang type, the *L*^*∗*^ value of the whole face and forehead and the *b*^*∗*^ value of the forehead and nose of the Cold-prescription group were larger than those of the Heat-prescription group (*p* = 0.023, 0.013, 0.004, and 0.001, resp.).

After the effects of the age, BMI, and systolic blood pressure on the complexion have been eliminated, significant differences were observed in the complexion according to the Cold- and Heat-prescription groups in each Sasang constitution. However, there was no significant difference in the male group as a whole.

Table 4 shows the facial complexion analysis for females. For the Tae-Eum type, the *L*^*∗*^ values of the forehead and nose of the Heat-prescription group were greater than those of the Cold-prescription group (*p* = 0.006 and 0.006, resp.). In contrast to the males, the *a*^*∗*^ value of the nose in the Cold-prescription group was greater than that of the Heat-prescription group (*p* = 0.034). For the So-Yang type, the *L*^*∗*^ values of the whole face, forehead, and nose of the Cold-prescription group were greater than those of the Heat-prescription group (*p* = 0.041, 0.011, and 0.042, resp.). However, there were no significant differences in the value of *L*^*∗*^*a*^*∗*^*b*^*∗*^ in complexion when Sasang constitution was not taken into consideration.

Although fewer differences were observed for females than for males, differences in the complexion were found according to the Cold- and Heat-prescription groups, despite the correction for the covariates of the age, BMI, and systolic blood pressure.

## 4. Discussion

Using the color space of *L*^*∗*^*a*^*∗*^*b*^*∗*^, we analyzed the differences in the complexion according to the Cold- and Heat-prescription groups and obtained interesting results. No significant difference was observed in the complexion according to the Cold- and Heat-prescription groups for the overall male and female groups consisting of the Tae-Eum, So-Eum, and So-Yang types, but significant differences were found for each Sasang constitution. To date, differences according to Sasang constitution have been the primary focus of studies [16, 17], but our results show differences within the same Sasang constitution. In addition, the results were consistent with the TKM literature.

Firstly, in the “Longevity and Life Preservation in Oriental Medicine,” for the Tae-Eum type, people with a blue and white facial color are stated to have fewer dryness syndromes, and people with a yellow, red, and black facial color have increased dryness syndromes. Thus, according to the literature, the Heat-pattern prescription group of the Tae-Eum type will often have a yellow, red, and black face, and the Cold-prescription group of the Tae-Eum type will have a blue or white face. In the analysis of this study, the *a*^*∗*^ value of the Heat-prescription group of the Tae-Eum type was significantly higher in males, and the complexion appeared red; however, this was not the case for females. Although not significant, the *b*^*∗*^ value of the Heat-prescription group of the Tae-Eum type was slightly higher for both sexes, and the color was yellowish.

The complexion may show the opposite tendency depending on the Sasang constitution. In the “Donguisusebowon Sasangchobonguan” [18], the complexion in healthy and sick individuals is different according to the Sasang constitution (“the So-Eum type is healthy when the complexion is soft violet, but ill when it is turbid yellow. In the case of the Tae-Eum type, it is healthy when the complexion is shiny violet. The So-Yang type is healthy when the complexion is shiny blue, but it is ill when it is white or black. In the case of the Tae-Yang type, it is healthy when the complexion is soft white but ill when the complexion is black”). In other words, the mechanism by which the physiological state is expressed as a facial color may vary depending on the Sasang constitution. In the analysis of this study, we also observed that the complexion of the Tae-Eum type and So-Yang type had opposite tendencies. The Tae-Eum type had a higher *L*^*∗*^ value in the Heat-prescription group and showed a bright complexion. In contrast, the So-Yang type had a higher *L*^*∗*^ value in the Cold-prescription group. Additionally, the *a*^*∗*^ and *b*^*∗*^ values differed by sex, but the two Sasang constitutions showed opposite tendencies.

According to the Sasang medical literature, the complexion is also different between the So-Eum and Tae-Eum types. In the “Donguisusebowon Sasangchobonguan” [18], when the face is oily, illness is thought to be worsened in the So-Eum type; however, this characteristic indicates improvement in illness in the Tae-Eum type. The analysis of this study confirmed that the complexions of the Tae-Eum and So-Eum types were not opposite but showed different tendencies. As described above, even in the case of the same Cold-prescription and Heat-prescription groups, the mechanism through which the complexion is expressed by the Sasang constitution is different; therefore, the analysis did not show any significant difference among the three Sasang constitutions.

In TKM, a person in the Cold-pattern group is characterized by sensitivity to cold and weak digestive power, whereas a person in the Heat-pattern group is characterized by sensitivity to heat and strong digestive power [1, 19]. Even if the Sasang constitution is different, the Cold- and Heat-pattern groups can be consistently classified according to clinical characteristics [20]. It is worthwhile to note that, unlike clinical symptoms, a significant difference was found in the analysis of the complexion only when the Sasang constitution was divided. It can be speculated that the factors related to Cold or Heat will affect the blood flow rate [21] and that various physiological functions will result in differences in the complexion. However, this study showed differences in the complexion related to the Sasang constitution as well as to the Cold-Heat pattern.

The Cold-Heat pattern is an important concept with high clinical value in TKM. The objectification and quantification of the complexion, a key diagnostic factor in determining the Cold-Heat pattern, are essential for the standardization of the diagnoses of TKM. This study showed a difference in the complexion among the Cold- and Heat-prescription groups by an objective method based on actual clinical data that will help determine the prescription. If larger scale clinical studies show the same results in the future, it will be possible to standardize diagnosis using the complexion. This will greatly contribute to enhancing the level of clinical medicine in TKM.

## 5. Conclusions

The complexion is an important diagnostic factor in Korean medicine and is related to the Sasang constitution. Based on actual clinical data, we found significant differences in the complexion between the Cold-prescription and Heat-prescription groups, which showed different patterns according to the Sasang constitution. The results confirmed that when adjusted for the age, BMI, and systolic blood pressure, the Cold-Heat pattern in TKM is related to the complexion.

## Figures and Tables

**Table 1:** Cold-Heat prescription and composition.

Sasang type	Cold and Heat prescription	Name of prescription	Prescription composition	Males	Females
TE type	Cold prescription	Taeumjowi-tang	Coicis Semen, Castaneae Semen, Raphani Semen, Schisandrae Fructus, Liriopis seu Ophiopogonis Tuber, Acori Graminei Rhizoma, Platycodonis Radix, Ephedrae Herba	11	21
		Choweseuncheng-tang	Coicis Semen, Castaneae Semen, Raphani Semen, Ephedrae Herba, Platycodonis Radix, Liriopis seu Ophiopogonis Tuber, Schisandrae Fructus, Acori Graminei Rhizoma, Polygalae Radix, Asparagi Tuber, Zizyphi Semen, Longan Arillus	17	28

TE type	Heat prescription	Galgeunhaegi-tang	Puerariae Radix, Scutellariae Radix, Ligustici Tenuissimi Rhizoma et Radix, Platycodonis Radix, Cimicifugae Rhizoma, Angelicae Dahuricae Radix	9	9
		Yuldahanso-tang	Puerariae Radix, Scutellariae Radix, Ligustici Tenuissimi Rhizoma et Radix, Raphani Semen, Platycodonis Radix, Cimicifugae Rhizoma, Angelicae Dahuricae Radix	33	61

SE type	Cold prescription	Jeokbaekhaogwanjung-tang	Cynanchi Wilfordii Radix, Polygoni Multiflori Radix, Alpiniae Officinari Rhizoma, Zingiberis Rhizoma, Citri Unshius Pericarpium, Citri Unshius Pericarpium Immaturus, Cyperi Rhizoma, Alpiniae Oxyphyllae Fructus, Zizyphi Fructus	2	9
		Gwankeibujalijung-tang	Ginseng Radix, Atractylodis Rhizoma Alba, Zingiberis Rhizoma, Cinnamomi Cortex, Paeoniae Radix, Citri Unshius Pericarpium, Glycyrrhizae Radix et Rhizoma, Aconiti Lateralis Radix Preparata	6	12
		Baekhaolijung-tang	Cynanchi Wilfordii Radix, Atractylodis Rhizoma Alba, Paeoniae Radix, Cinnamomi Ramulus, Zingiberis Rhizoma, Citri Unshius Pericarpium, Glycyrrhizae Radix et Rhizoma	1	2
		Osuyubujalijung-tang	Ginseng Radix, Atractylodis Rhizoma Alba, Zingiberis Rhizoma, Cinnamomi Cortex, Paeoniae Radix, Citri Unshius Pericarpium, Glycyrrhizae Radix et Rhizoma, Evodiae Fructus, Foeniculi Fructus, Psoraleae Semen, Aconiti Lateralis Radix Preparata	0	1
		Hyangsayangwi-tang	Ginseng Radix, Atractylodis Rhizoma Alba, Paeoniae Radix, Glycyrrhizae Radix et Rhizoma, Pinelliae Tuber, Cyperi Rhizoma, Citri Unshius Pericarpium, Zingiberis Rhizoma, Crataegi Fructus, Amomi Fructus, Amomi Fructus Rotundus, Perillae Folium, Zizyphi Fructus	1	6

SE type	Heat prescription	Palmulgunja-tang	Ginseng Radix, Astragali Radix, Atractylodis Rhizoma Alba, Paeoniae Radix, Angelicae Gigantis Radix, Cnidii Rhizoma, Citri Unshius Pericarpium, Glycyrrhizae Radix et Rhizoma, Perillae Folium, Zizyphi Fructus	31	38
		Bojungikgi-tang	Ginseng Radix, Astragali Radix, Glycyrrhizae Radix et Rhizoma, Atractylodis Rhizoma Alba, Angelicae Gigantis Radix, Citri Unshius Pericarpium, Pogostemonis Herba, Perillae Folium, Zizyphi Fructus	3	0

SY type	Cold prescription	Hyungbangsabaek-san	Rehmanniae Radix Recens, Poria Sclerotium, Alismatis Rhizoma, Anemarrhenae Rhizoma, Gypsum Fibrosum, Osterici seu Notopterygii Radix et Rhizoma, Araliae Continentalis Radix, Schizonepetae Spica, Saposhnikoviae Radix	9	16
		Hyeongbangdojeok-san	Rehmanniae Radix Recens, Akebiae Caulis, Scrophulariae Radix, Trichosanthis Semen, Peucedani Radix, Osterici seu Notopterygii Radix et Rhizoma, Araliae Continentalis Radix, Schizonepetae Spica, Saposhnikoviae Radix	3	31
		Hyeongbangjihwang-tang	Rehmanniae Radix Preparata, Corni Fructus, Poria Sclerotium, Alismatis Rhizoma, Plantaginis Semen, Osterici seu Notopterygii Radix et Rhizoma, Araliae Continentalis Radix, Schizonepetae Spica, Saposhnikoviae Radix	21	86

SY type	Heat prescription	Yanggyeoksanhwa-tang	Rehmanniae Radix Recens, Lonicerae Folium et Caulis, Forsythiae Fructus, Gardeniae Fructus, Menthae Herba, Anemarrhenae Rhizoma, Gypsum Fibrosum, Saposhnikoviae Radix, Schizonepetae Spica	28	33

**Table 2:** General characteristics of the subjects.

	TE type			SE type			SY type		
	CG	HG	*p* value	CG	HG	*p* value	CG	HG	*p* value
Males									
*N*	28	42		10	34		33	28	
Age	45.11 ± 2.79	47.74 ± 2.55	0.499	43.38 ± 4.39	43.10 ± 2.78	0.962	43.32 ± 2.40	49.97 ± 2.86	0.078
Height	172.37 ± 1.15	171.57 ± 1.08	0.622	168.93 ± 2.03	171.1 ± 0.98	0.310	172.49 ± 1.13	171.03 ± 1.26	0.390
Weight	77.76 ± 1.70	76.26 ± 1.52	0.520	60.86 ± 1.64	63.70 ± 1.40	0.200	70.62 ± 1.43	70.72 ± 1.69	0.962
BMI	26.24 ± 0.63	25.86 ± 0.40	0.594	21.41 ± 0.79	21.76 ± 0.44	0.706	23.73 ± 0.41	24.16 ± 0.50	0.505
Females									
*N*	49	70		30	38		133	33	
Age	44.78 ± 2.04	47.09 ± 1.78	0.399	49.55 ± 2.82	39.34 ± 2.44	0.008^*∗∗*^	44.28 ± 1.27	53.20 ± 2.66	0.002^*∗∗*^
Height	160.08 ± 0.76	159.75 ± 0.61	0.734	157.31 ± 1.10	159.71 ± 0.96	0.105	158.23 ± 0.50	157.02 ± 0.93	0.274
Weight	64.10 ± 0.99	65.26 ± 0.98	0.422	49.58 ± 1.10	53.91 ± 1.01	0.005^*∗∗*^	54.60 ± 0.64	55.33 ± 1.14	0.604
BMI	25.04 ± 0.38	25.60 ± 0.39	0.319	20.07 ± 0.44	21.15 ± 0.37	0.061	21.81 ± 0.25	22.44 ± 0.43	0.251

Data are presented in means ± standard error. CG, Cold-prescription group; HG, Heat-prescription group. ^*∗∗*^*p* < 0.01. The analysis of statistical data was performed using *t*-test.

**Table 3:** Facial complexion for males according to Cold- and Heat-prescription group.

Region	*L* ^*∗*^ *a* ^*∗*^ *b* ^*∗*^ color	Group	All males		Tae-Eum type		So-Eum type		So-Yang type	
			Mean ± SE	*p* value	Mean ± SE	*p* value	Mean ± SE	*p* value	Mean ± SE	*p* value
Total	*L* ^*∗*^	CG	58.99 ± 0.37	0.666	58.29 ± 0.53	0.151	59.84 ± 1.13	0.942	58.95 ± 0.53	0.023^*∗*^
		HG	58.78 ± 0.31		59.28 ± 0.43		59.94 ± 0.61		57.08 ± 0.58	
	*a* ^*∗*^	CG	13.98 ± 0.21	0.192	14.18 ± 0.33	0.045^*∗*^	12.65 ± 0.52	0.128	14.40 ± 0.27	0.264
		HG	14.34 ± 0.18		15.06 ± 0.27		13.57 ± 0.28		13.93 ± 0.30	
	*b* ^*∗*^	CG	19.62 ± 0.21	0.838	19.20 ± 0.32	0.307	19.00 ± 0.52	0.049^*∗*^	20.09 ± 0.33	0.072
		HG	19.68 ± 0.18		19.63 ± 0.26		20.21 ± 0.28		19.17 ± 0.36	

Forehead	*L* ^*∗*^	CG	63.52 ± 0.47	0.959	62.91 ± 0.72	0.694	63.66 ± 1.46	0.191	63.66 ± 0.61	0.013^*∗*^
		HG	63.49 ± 0.40		63.28 ± 0.59		65.88 ± 0.79		61.31 ± 0.66	
	*a* ^*∗*^	CG	13.18 ± 0.26	0.209	13.22 ± 0.04	0.016^*∗*^	11.73 ± 0.67	0.188	13.82 ± 0.34	0.117
		HG	13.62 ± 0.22		14.51 ± 0.33		12.75 ± 0.36		13.00 ± 0.37	
	*b* ^*∗*^	CG	20.66 ± 0.26	0.858	20.20 ± 0.40	0.680	19.86 ± 0.63	0.018^*∗*^	21.33 ± 0.37	0.004^*∗∗*^
		HG	20.60 ± 0.22		20.41 ± 0.32		21.62 ± 0.34		19.64 ± 0.41	

Cheek	*L* ^*∗*^	CG	55.69 ± 0.36	0.515	55.03 ± 0.48	0.018^*∗*^	56.42 ± 1.12	0.433	55.67 ± 0.56	0.067
		HG	55.38 ± 0.31		56.53 ± 0.39		55.41 ± 0.60		54.08 ± 0.61	
	*a* ^*∗*^	CG	14.22 ± 0.20	0.233	14.57 ± 0.32	0.166	13.10 ± 0.50	0.205	14.41 ± 0.27	0.730
		HG	14.54 ± 0.17		15.15 ± 0.26		13.84 ± 0.27		14.27 ± 0.30	
	*b* ^*∗*^	CG	18.98 ± 0.22	0.549	18.65 ± 0.32	0.182	18.53 ± 0.54	0.201	19.27 ± 0.37	0.617
		HG	19.15 ± 0.18		19.20 ± 0.26		19.32 ± 0.29		18.99 ± 0.40	

Nose	*L* ^*∗*^	CG	63.14 ± 0.45	0.558	62.31 ± 0.71	0.750	64.97 ± 1.36	0.716	62.96 ± 0.56	0.028
		HG	62.79 ± 0.38		62.01 ± 0.58		65.54 ± 0.73		61.05 ± 0.61	
	*a* ^*∗*^	CG	15.60 ± 0.28	0.177	15.68 ± 0.46	0.019^*∗*^	13.68 ± 0.70	0.065	16.38 ± 0.36	0.063
		HG	16.11 ± 0.24		17.13 ± 0.38		15.19 ± 0.38		15.35 ± 0.39	
	*b* ^*∗*^	CG	19.53 ± 0.25	0.488	18.85 ± 0.36	0.406	18.39 ± 0.74	0.051	20.36 ± 0.35	0.001^*∗∗*^
		HG	19.30 ± 0.21		19.24 ± 0.29		20.09 ± 0.40		18.54 ± 0.38	

Total is the average *L*^*∗*^*a*^*∗*^*b*^*∗*^ value calculated using the *L*^*∗*^*a*^*∗*^*b*^*∗*^ value of three regions (forehead, cheek, and nose). SE, standard error; CG, Cold-prescription group; HG, Heat-prescription group. ^*∗*^*p* < 0.05;  ^*∗∗*^*p* < 0.01. The analysis of statistical data was performed using ANCOVA. The mean and *p* value were adjusted by covariates (age, BMI, and systolic blood pressure).

**Table 4:** Facial complexion for females according to Cold- and Heat-prescription groups.

Region	*L* ^*∗*^ *a* ^*∗*^ *b* ^*∗*^ color	Group	All females		Tae-Eum type		So-Eum type		So-Yang type	
			Mean ± SE	*p* value	Mean ± SE	*p* value	Mean ± SE	*p* value	Mean ± SE	*p* value
Total	*L* ^*∗*^	CG	63.83 ± 0.21	0.848	63.83 ± 0.49	0.157	64.66 ± 0.58	0.137	63.65 ± 0.23	0.041^*∗*^
		HG	63.89 ± 0.26		64.76 ± 0.41		63.42 ± 0.51		62.55 ± 0.48	
	*a* ^*∗*^	CG	11.50 ± 0.15	0.797	12.47 ± 0.25	0.096	11.01 ± 0.38	0.350	11.19 ± 0.20	0.207
		HG	11.44 ± 0.18		11.92 ± 0.21		10.51 ± 0.33		11.79 ± 0.42	
	*b* ^*∗*^	CG	19.71 ± 0.19	0.513	20.34 ± 0.36	0.322	20.07 ± 0.51	0.321	19.36 ± 0.24	0.287
		HG	19.90 ± 0.23		20.82 ± 0.30		19.34 ± 0.45		18.76 ± 0.50	

Forehead	*L* ^*∗*^	CG	68.00 ± 0.29	0.109	67.92 ± 0.68	0.006^*∗∗*^	68.99 ± 0.78	0.553	67.73 ± 0.31	0.011^*∗*^
		HG	68.74 ± 0.35		70.42 ± 0.57		68.33 ± 0.68		65.93 ± 0.62	
	*a* ^*∗*^	CG	10.34 ± 0.16	0.585	11.12 ± 0.29	0.067	9.71 ± 0.41	0.740	10.13 ± 0.22	0.232
		HG	10.20 ± 0.19		10.43 ± 0.24		9.52 ± 0.36		10.73 ± 0.45	
	*b* ^*∗*^	CG	20.48 ± 0.22	0.416	21.30 ± 0.43	0.243	20.90 ± 0.63	0.345	20.00 ± 0.27	0.325
		HG	20.76 ± 0.27		21.96 ± 0.36		20.04 ± 0.55		19.38 ± 0.56	

Cheek	*L* ^*∗*^	CG	60.70 ± 0.20	0.176	61.01 ± 0.46	0.713	61.01 ± 0.54	0.051	60.56 ± 0.25	0.179
		HG	60.26 ± 0.25		60.79 ± 0.38		59.50 ± 0.47		59.79 ± 0.51	
	*a* ^*∗*^	CG	12.19 ± 0.15	0.924	13.19 ± 0.26	0.240	11.93 ± 0.38	0.168	11.82 ± 0.20	0.179
		HG	12.22 ± 0.18		12.79 ± 0.21		11.18 ± 0.33		12.44 ± 0.41	
	*b* ^*∗*^	CG	19.33 ± 0.18	0.645	19.82 ± 0.35	0.395	19.63 ± 0.48	0.332	19.07 ± 0.24	0.264
		HG	19.46 ± 0.22		20.21 ± 0.29		18.97 ± 0.42		18.46 ± 0.49	

Nose	*L* ^*∗*^	CG	67.71 ± 0.30	0.098	67.26 ± 0.69	0.006^*∗∗*^	69.91 ± 0.86	0.205	67.39 ± 0.31	0.042^*∗*^
		HG	68.50 ± 0.37		69.77 ± 0.57		68.36 ± 0.75		65.92 ± 0.64	
	*a* ^*∗*^	CG	11.81 ± 0.18	0.423	13.05 ± 0.33	0.034^*∗*^	10.77 ± 0.46	0.536	11.48 ± 0.25	0.163
		HG	11.58 ± 0.22		12.12 ± 0.28		10.36 ± 0.41		12.29 ± 0.51	
	*b* ^*∗*^	CG	19.04 ± 0.20	0.354	19.96 ± 0.39	0.460	19.28 ± 0.59	0.505	18.57 ± 0.24	0.464
		HG	19.33 ± 0.24		20.35 ± 0.33		18.72 ± 0.52		18.17 ± 0.50	

Total is the average *L*^*∗*^*a*^*∗*^*b*^*∗*^ value calculated using the *L*^*∗*^*a*^*∗*^*b*^*∗*^ value of three regions (forehead, cheek, and nose). SE, standard error; CG, Cold-prescription group; HG, Heat-prescription group. ^*∗*^*p* < 0.05;  ^*∗∗*^*p* < 0.01. The analysis of statistical data was performed using ANCOVA. The mean and *p* value were adjusted by covariates (age, BMI, and systolic blood pressure).
